# First Trial of a Novel Caseous Lymphadenitis Inactivated Vaccine in South Korea: Experimental Evaluation across Various Animal Models

**DOI:** 10.3390/pathogens13090729

**Published:** 2024-08-28

**Authors:** Gyeong-Seo Park, Somin Lee, Min A Jeong, So Young Lee, Dong-Geun Cho, WonIl Cho, Seung-Chai Kim, Hwan-Ju Kim, Taek Geun Lee, Chang-Gi Jeong, Won-Il Kim, Yeonsu Oh, Ho-Seong Cho, Byoung-Joo Seo, Chonghan Kim

**Affiliations:** 1 Vaccine Lab, WOOGENE B&G Co., Ltd., Seoul 07299, Republic of Korea; gyeoungseopark@gmail.com (G.-S.P.); somin94@woogenebng.com (S.L.); jm0613@woogenebng.com (M.A.J.); charleesy808@gmail.com (S.Y.L.); 2 Osan Black Goat Farm, Gokseong 57518, Republic of Korea; dkcho55@naver.com (D.-G.C.); planet276@naver.com (W.C.); 3 College of Veterinary Medicine, Bio-Safety Research Institute, Jeonbuk National University, Iksan 54596, Republic of Korea; leesor2@jbnu.ac.kr (S.-C.K.); felix1116@jbnu.ac.kr (H.-J.K.); shortm@naver.com (T.G.L.); jcg0102@gmail.com (C.-G.J.); kwi0621@jbnu.ac.kr (W.-I.K.); 4 College of Veterinary Medicine and Institute of Veterinary Science, Kwangwon National University, Chuncheon 24341, Republic of Korea; yeonoh@kangwon.ac.kr

**Keywords:** *Corynebacterium pseudotuberculosis*, Korean Native Black Goats (KNBGs), Caseous Lymphadenitis (CLA), inactivated vaccine, animal trials

## Abstract

Caseous lymphadenitis (CLA) is a chronic and subclinical bacterial disease of ruminants caused by *Corynebacterium pseudotuberculosis* (*C. pseudotuberculosis*) infection. Until 2014, there were no reports of CLA outbreaks in South Korea; however, the prevalence of CLA cases has steadily increased. In this study, we used recently obtained field isolates to develop the first inactivated CLA vaccine in South Korea and evaluated it in various animal models. The inactivated vaccine was evaluated for virulence and effectiveness. Mice were tested for virulence and immunization challenges, and guinea pigs and Korean Native Black Goats (KNBGs) evaluated various vaccine concentrations to determine the optimal dose and effectiveness. In the case of KNBGs, clinical symptoms were not observed after vaccination. In addition, CLA-specific IgG was detected at a significantly (*p* < 0.05) high level and was maintained. In histopathological evaluations, inflammation was predominantly observed in the prefemoral lymph nodes in the non-vaccinated+CHAL group. The genetic diversity of *C. pseudotuberculosis*, which has become widespread in South Korea, is less than 0.5% our vaccine is expected to prevent infection by a wide range of strains effectively. In summary, our CLA vaccine can potentially prevent CLA and foster the growth of South Korea’s domestic KNBG industry.

## 1. Introduction

Caseous lymphadenitis (CLA) is a chronic, pyogenic, and subclinical bacterial disease of goats and sheep. CLA is widespread worldwide but more prevalent in areas where intensive husbandry is practiced [1,2]. The goat and sheep industry, which includes ruminants, is associated with significant economic losses due to the slaughtering of infected animals and reduced wool production [3,4]. In previous studies, CLA has been found to affect approximately 64 countries, and in Asia, approximately 11 countries are affected [5]. Australia has been reported to have the highest CLA prevalence at 61%, with a prevalence of approximately 30% or more reported for other countries [6,7,8]. Before 2014, there were no reports of outbreaks in Korea. However, according to the Animal and Plant Quarantine Agency (APQA) report, approximately 50% of cases were reported nationwide regardless of the region in Korea.

The causal agent of CLA is *Corynebacterium pseudotuberculosis* (*C. pseudotuberculosis*), a facultative, intracellular, non-capsulated, nonmotile, fimbriated, gram-positive bacterium [9,10]. *C. pseudotuberculosis* is characterized by two major virulence factors. One is a mycolic acid-rich cell wall, and the other is a potent phospholipase-D (PLD) exotoxin [11]. Among the toxins, the most studied is the PLD toxin protein. PLDs are exotoxins that increase local vascular permeability and serve to spread the pathogen from the original infection site to the host [12,13]. The role of PLD as a crucial virulence factor in the development of CLA has been demonstrated, particularly in the dissemination of bacteria from the primary infection site to other organs, such as the lungs and mesenteric lymph nodes [14]. Due to *C. pseudotuberculosis* infection, CLA can occur in external or visceral forms, either separately or together [15,16]. In general, external CLA lesions first appear as abscesses and later transform into abscess-like onion appearances, which are located within the superficial lymph nodes [17]. And also, some CLA-infected animals generally exhibit orchitis, cellulitis, and mastitis [18,19,20].

To eradicate the spread of CLA, many studies have attempted to develop an effective vaccine to prevent CLA infection. The majority of the vaccine development studies were conducted based on formalin-inactivated vaccines. The commercialized CLA vaccine is based on traditional vaccine technology, including toxoid vaccines, bacterins, and live attenuated bacteria [21,22]. However, most studies have focused on CLA-specific IgG and IFN-γ as immunological markers for high-quality CLA vaccines [23,24]. During the past decade, genetic variations in field strains have been observed. Since its initial discovery in the 2010s, field strains isolated in Korea have exhibited distinct genetic characteristics compared to foreign field strains. However, due to the unavailability of CLA preventive vaccines in Korea, it spread nationwide.

Accordingly, the primary objective of this study was to formulate an inactivated vaccine against CLA. To achieve this goal, strains of *C. pseudotuberculosis* were sourced from local strains in Korea, and vaccine candidates were carefully generated through systematic evaluation processes. Consequently, an inactivated vaccine was successfully developed. Substantial experimentation ensued, encompassing rigorous safety and efficacy assessments, thereby substantiating its potential as a viable strategy for combating CLA outbreaks.

## 2. Materials and Methods

### 2.1. Bacterial Strains and Culture Conditions

The bacterial strains used in the study are listed in Table A1. In a previous study [25], 74 isolates of *C. pseudotuberculosis* from Korean native black goats (KNBGs, *Capra hiscus coreanae*) were evaluated. Based on a preliminary screening of molecular and bacterial characteristics, as well as multilocus sequence typing (MLST) analysis and an evaluation of bacterial proliferation among field isolates, WOOGENE B and G-*C. Pseudotuberculosis* (WGB-Cory, [Master cell bank]) was selected as the vaccine candidate strain due to its superior bacterial proliferation rate and viability than the other field isolates. The vaccine candidate was cultured on Brain Heart Infusion (BHI, Difco^TM^, Becton, Dickinson and Company, Franklin Lakes, NJ, USA) agar medium for 48 h at 37 °C. The bacterial colonies were confirmed for purity and subcultured on BHI broth medium supplemented with 0.5% Tween^®^ 80 (Polysorbate 80, Sigma-Aldrich, Darmstadt, Germany).

### 2.2. DNA Extraction and Identification of Bacteria

WGB-Cory colonies were separately cultured on a BHI broth medium supplemented with 0.5% Tween^®^ 80 for 48 h at 37 °C for bacterial DNA extraction. Bacterial DNA was extracted using a QIAamp DNA Mini Kit (Qiagen, Hilden, Germany) following the manufacturer’s instructions. The extracted bacterial DNA was quantified using a Qubit DNA Quantification method. To evaluate the vaccine candidate, PLD genes were identified by PCR using a specific primer set (Table 1) according to a previous study [25]. The PCR products were visualized and analyzed on a 1.5% agarose gel with RedSafe^TM^ (Sigma-Aldrich, Darmstadt, Germany).

### 2.3. Detection of Virulence Genes

Seventeen DNA sequences of PLD-positive *C. pseudotuberculosis* virulence genes, including oligopeptide permease protein-related genes (OppA, OppB, OppC, OppD, and OppF), integral membrane protein (FagA), iron enterobactin transporter (FagB), ATP binding cytoplasmic membrane protein (FagC), iron siderophore binding protein (FagD), sigma factors E (SigE), tip protein C (SpaC), zinc-dependent superoxide dismutase (SodC), protein kinase G (PknG), neuraminidase (NanH), glutaredoxin-like protein (NrdH), copper resistance protein (CopC), and Pilus assembly protein (CpaE) were detected by PCR with primers [26,27] synthesized by Cosmogenetech Co., Ltd. (Seoul, Republic of Korea). The PCR amplification conditions were as follows: initial denaturation for 5 min at 94 °C, followed by 30 cycles of 94 °C for 40 s, 61 °C for 40 s (58 °C for FagA, 55 °C for FagB, SigE, SpaC, SodC, PknG, NanH, OppA, OppB, OppC, OppD, OppF, CopC, NrdH, and CpaE, 60 °C for FagC), and 72 °C for 40 s, with a final elongation of 72 °C for 10 min [26,27].

### 2.4. 16S rRNA Amplification and Sequencing

The 16S rRNA gene was amplified under the following conditions: 5 min of initial denaturation at 94 °C; 30 cycles at 94 °C for 30 s, 54 °C for 30 s, and 72 °C for 90 s; and 10 min of final elongation at 72 °C [26]. The final PCR product was subsequently sequenced by Cosmogenetech Co., Ltd. (Seoul, Republic of Korea). The obtained 16S rRNA sequence was subjected to online alignment with the BLAST program. A phylogenic tree was constructed by the neighbor-joining method using the same software. Phylogenetic trees were generated by the maximum likelihood method via the Kimura two-parameter (K2P) model with a gammadistribution and invariant sites (K2P + G4 + I) using MEGA (ver. 11). The 16S rRNA sequences of the WGB-Cory were used as an outgroup in the phylogenetic analyses. Support for specific genotypes of the *C. pseudotuberculosis* was determined with 1000 bootstrap replicates (≥70%).

### 2.5. Vaccine Formulations

For safety and efficacy testing, three inactivated vaccines (F1 to F3) were manufactured and compared. Briefly, the three vaccine candidates were produced by culturing *C. pseudotuberculosis*, inactivating it with a 2.0% formaldehyde solution (Sigma-Aldrich, USA) at 37 °C for 24 h, and then subjecting the inactivated bacterial cultures to high-speed centrifugation to obtain a pellet. The pellets containing the whole inactivated bacterin were resuspended in phosphate-buffered saline (PBS, 0.01 M, pH 7.4), and this suspension was then formulated with 10% carbopol (Carbopol 934 polymer, Lubrizol, Wickliffe, OH, USA) and 0.1% saponin (Junsei Co., Tokyo, Japan) following the manufacturer’s instructions. The three inactivated vaccines contained the following antigen concentrations: F1, 1.0 × 10^7^ colony-forming units (CFU)/mL; F2, 1.0 × 10^6^ CFU/mL; and F3, 1.0 × 10^5^ CFU/mL, respectively. The inactivation of the vaccines was confirmed if no bacterial growth was observed 48 h after plating 100 uL of the cultured sample on BHI agar medium. The physical properties and sterility of the inactivated vaccines were evaluated according to the release criteria for veterinary medicinal products set by the Animal and Plant Quarantine Agency (APQA) of the Republic of Korea.

### 2.6. Animal Studies

The animal experiments were performed on 70 mice, 10 guinea pigs, and 25 Korean Native black goats. The animal experiment protocol was approved by the Jeonbuk National University Institutional Animal Care and Use Committee (approval number 2022-032) and performed following the guidelines and regulations detailed by the committee.

#### 2.6.1. Mice

To evaluate the comparative virulence of different challenge concentrations of *C. pseudotuberculosis* isolates, LD_50_ tests were performed. Forty 4-week-old female BALB/c mice were purchased from Samtako Co., Ltd. (Osan-si, Gyeonggi-Do, Republic of Korea) and randomly divided into 4 groups (10 animals each group); Group A (10^8^ CFU/mL), Group B (10^7^ CFU/mL), Group C (10^6^ CFU/mL), and Group D (10^5^ CFU/mL); vaccine concentrations, respectively. After 7 days of acclimatization, all groups were inoculated intraperitoneally (IP) with 0.2 mL of *C. pseudotuberculosis* isolate. The mice were monitored twice daily over 10 days, and the numbers of living and deceased mice were recorded.

For the immunization and challenge experiments, thirty 4-week-old female BALB/c mice were purchased from Samtako Co., LTD. (Osan-si, Gyeonggi-Do, Republic of Korea) and randomly divided into 3 groups (10 animals each group); Group 1 (vaccinated group), Group 2 (non-vaccinated+CHAL; non-vaccinated and challenge), and Group 3 (non-vaccinated+CHAL; non-vaccinated and non-challenge). After 7 days of acclimatization, the safety and efficacy of the vaccine were performed. Group 1 was subcutaneously (SC) inoculated with 0.2 mL of inactivated vaccine (10^6^ CFU/mL) at 0 DPV (days post-vaccination) and inoculated with a booster at 14 DPV. At 28 DPV, Group 1 and Group 2 were intraperitoneally inoculated with 0.2 mL of *C. pseudotuberculosis* field isolate (10^6^ CFU/mL). The animals in Group 3 remained uninfected. The condition of the mice was recorded every day.

#### 2.6.2. Guinea Pigs

To identify the sufficient concentration of the vaccine, fifty 5-week-old female Hartley Guinea pigs were purchased from Samtako Co., Ltd. and randomly divided into 5 groups (10 animals each group); Group A (vaccine group 1; F1), Group B (vaccine group 2; F2), Group C (vaccine group 3; F3), Group D (non-vaccinated+CHAL group; non-vaccinated, challenged), and Group E (non-vaccinated+nonCHAL group; phosphate-buffered saline (PBS, 0.01 M, pH 7.4), non-vaccinated and non-challenged). After 3 days of acclimatization, Groups A, B, and C were intramuscularly (IM) inoculated with the inactivated *C. pseudotuberculosis* vaccine containing different concentrations at 0 and 14 DPV. To conduct a comparative evaluation of the optimal vaccine concentration, Group D was inoculated with *C. pseudotuberculosis*. Group E remained until the end of the experiment. The condition of the guinea pigs was recorded until the end of the experiment. Blood was collected on the designated dates to conduct the guinea pig serum titer test against *C. pseudotuberculosis*.

#### 2.6.3. Korean Native Black Goats (KNBGs)

**Figure 1:**
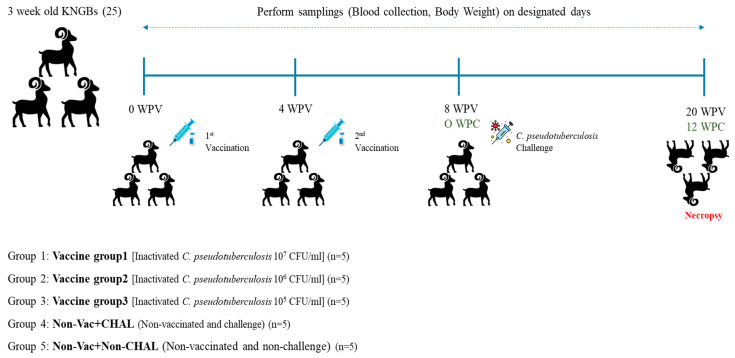
Overview of the animal experiment. Twenty-five 12-week-old CLA-seronegative KNBGs were randomly divided into five groups. Among the vaccine groups, vaccine group 1 (10^7^ CFU/mL), vaccine group 2 (10^6^ CFU/mL), and vaccine group 3 (10^5^ CFU/mL) were intramuscularly () inoculated with the inactivated vaccine on Day 1 and received a booster vaccination at 4 WPV. KNBGs were monitored daily until the end of the experiment for the development of clinical signs such as abnormal behavior and dyspnea. Blood samples were collected and measured by CLA-specific ELISA. Body weights of all KNBGs were measured at 0, 8, and 12 WPV, and the AWWG was measured at 8 and 12 WPV.

To evaluate the safety and efficacy of the vaccine in the target animal species, an experiment was conducted on a KNBG farm where CLA had previously occurred. The experiments were conducted with individuals who did not show clinical symptoms of CLA infection and who did not have CLA antibodies. Twenty-five, 12-week-old, CLA-seronegative KNBGs were randomly divided into 5 groups (each 5 animals in each group); vaccine group 1 (10^7^ CFU/mL), vaccine group 2 (10^6^ CFU/mL), vaccine group 3 (10^5^ CFU/mL), non-vaccinated (challenged), and non-vaccinated (non-challenged). An overview of the animal experiment is displayed in Figure 1. The vaccine groups were intramuscularly (I.M.) inoculated with the inactivated vaccine on 0 DPV and with a booster vaccination at 4 WPV (weeks postvaccination). After 4 WPV, vaccinated and non-vaccinated+CHAL groups were challenged with *C. pseudotuberculosis* with 10^6^ CFU/mL. All groups of KNBGs were monitored daily until the end of the experiment. Monitoring included the development of clinical signs such as abnormal behavior and dyspnea; the clinical score was 0 = normal, 1 = CLA-induced abscess identified, 2 = CLA-induced abscess enlarges, with hardness and hair loss around the abscess, 3 = CLA-induced abscess bursts, and 4 = death. Blood samples were collected at two-week intervals from all groups until the end of the experiment to evaluate the levels of CLA-specific antibodies. The body weights of all KNBGs were measured at 0, 8, and 12 WPV, and the average weekly weight gain (AWWG) was calculated at 8 and 12 WPV. All KNBGs were humanely euthanized at 20 WPV. Euthanasia was performed by electrocution after an intramuscular injection of 2.0 mL of azaperone (40 mg/mL, Stressnil, WOOGENE B and G Co., Ltd., Seoul, South Korea). During the necropsy, various lymph nodes were obtained for further analysis.

### 2.7. Anti-CLA-Specific Antibody Detection

The serum from animal experiment groups (guinea pigs, KNBGs) was tested for anti-PLD-specific antibody (IgG) using a commercially available ELISA kit (ELITEST CLA, HYPHEN BioMed, Neuville sur Oise, France) according to the manufacturer’s manual and previous study [28]. The ELITEST CLA is a commercial ELISA kit designed for enzyme immunoassay (EIA) to detect specific IgG antibodies against the causative agent of caseous lymphadenitis in sheep and/or goat serum. Especially for the analysis of guinea pig serum using the ELITEST CLA kit, different secondary antibodies were used: anti-guinea pig IgG HRP. The S/P ratio (ratio of the net optical density of the test sample to the net optical density of the positive control) was interpreted. Serum samples with an S/P ratio higher than 0.5 (>0.5) were considered positive for CLA-specific antibodies, while samples with an S/P ratio less than 0.2 (<0.2) were considered negative. Serum samples with an S/P ratio between 0.2 and 0.5 were retested for result analysis.

### 2.8. Quantification of Bacterial Load in Lymph Nodes

To quantify bacterial load, all KNBG groups were euthanized at the end of the experiment, and lymph nodes were collected. Lymph nodes were collected in eight sites: challenge site, prefemoral, retropharyngeal, submandibular, prescapular, popliteal, superficial inguinal, and genital lymph nodes. Each lymph node (3 g) was homogenized in 7 mL of PBS and centrifuged at 2500 rpm for 10 min to obtain the supernatant. The supernatant from homogenized lymph nodes was serially diluted 10-fold and spread on BHI agar medium plates at 37 °C. After 48 h of incubation, all BHI agar medium plates were evaluated to calculate CFUs.

### 2.9. Histopathological Evaluation in KNBG’s Lymph Nodes

Histopathological evaluation was conducted on the prescapular lymph nodes, which are close to the vaccination and challenge sites, and confirmed the presence of inflammatory responses, congestion, infiltration of neutrophils and macrophages, and hemorrhage induced by the vaccination [29]. For H and E staining, the tissue embedded on a glass slide was deparaffinized, rehydrated in distilled water, and then stained with Mayer’s hematoxylin for 1 min. After washing 4–5 times, the sections were decolorized with 0.5% alcohol, and then the slides were washed with 1× PBS for 1 min. Thereafter, the sections were counterstained with alcoholic eosin for 30 s and then dehydrated through 3 changes of 95% EtOH and 2 changes of 100% EtOH for 60 s. The sections were cleared with 3 changes of xylene for the 60 s and then mounted.

### 2.10. Data Analysis

Graphical representations of the data were prepared using GraphPad Prism 9.00 (GraphPad Software, San Diego, CA, USA), and the statistical analysis was performed using SPSS Advanced Statistics 17.0 software (SPSS, Inc., Chicago, IL, USA). Two-way ANOVA with Tukey’s multiple comparison tests was used to analyze the significance of variability within animal experimental groups for clinical data (clinical score), bacterial load, and anti-CLA antibodies. A nonparametric one-way ANOVA (Kruskal–Wallis test) was used to compare the AWWG. Differences considered statistically significant are indicated by asterisks and different letters over the bars.

## 3. Results

### 3.1. Characterization of the WGB-Cory: PCR Identification of Virulence Factors

**Figure 2:**
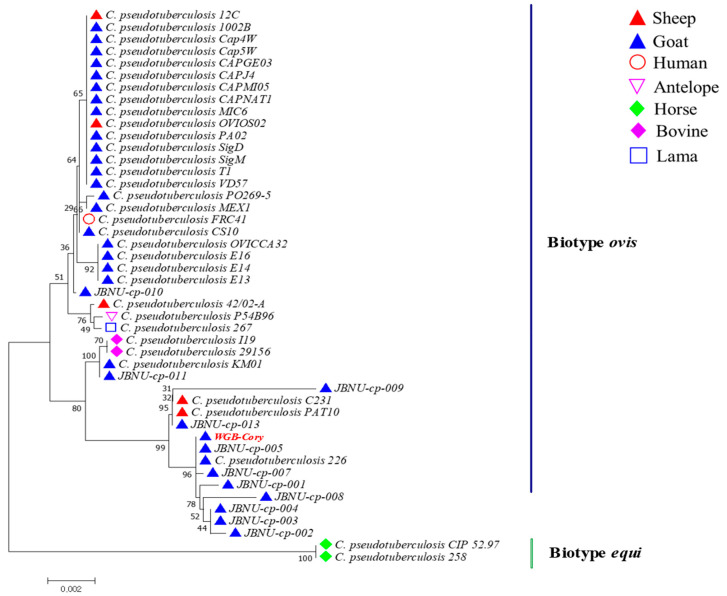
Phylogenetic tree depicting the evolutionary relationships within the *Corynebacterium pseudotuberculosis* WGB-Cory strain based on 16s RNA sequence data. The comprehensive genomic analysis provides insights into the composition and validation of the strain’s genome. The WGB-Cory, marked in red, is a vaccine strian and was highlighted to distinguish it from other strains.

PCR analysis of DNA extracted from 9 isolates of *C. pseudotuberculosis* confirmed the presence of 17 virulence factors: OppA, OppB, OppC, OppD, OppF, FagA, FagB, FagC, FagD, SigE, SpaC, SodC, PknG, NanH, NrdH, CopC, and CpaE (Table A1). The proliferation of bacteria was evaluated for nine isolates of *C. pseudotuberculosis* that were assessed for virulence genes. Among the nine isolates of *C. pseudotuberculosis*, 1 isolate of *C. pseudotuberculosis* exhibited exceptionally high proliferation capability, and we selected it as the vaccine candidate. The resulting phylogenetic tree, depicted in Figure 2, shows the evolutionary relationships between the vaccine candidate WGB-Cory and other *C. pseudotuberculosis* isolates. The phylogenetic analysis results confirmed that the vaccine candidate is included within the biotype *ovis* strains in the phylogenetic tree. In addition, the analysis showed low bootstrap values and intergroup divergence within biotype *ovis* strains. Based on the results of virulence genes and bacterial proliferation, the final selected vaccine candidate was evaluated for safety and efficacy in experimental animals and KNBGs.

### 3.2. Evaluation of the Pathogenicity and Safety of the Vaccine Candidates

**Figure 3:**
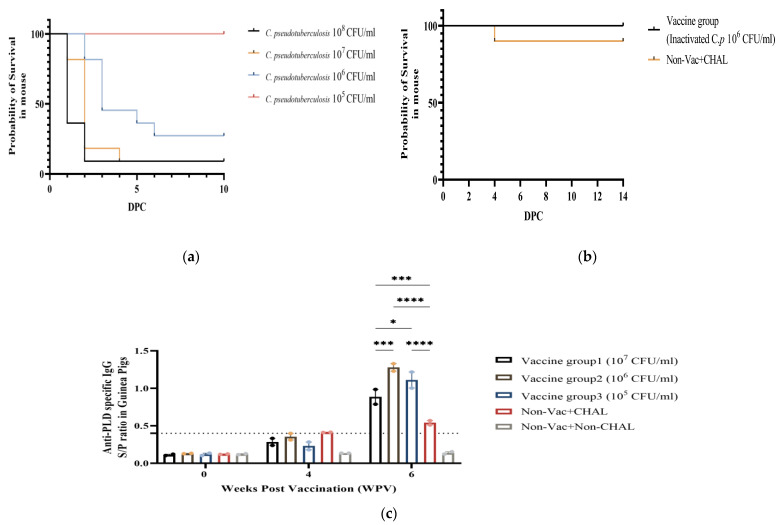
(**a**) In the LD_50_ experiment, mice exposed to different concentrations of *C. pseudotuberculosis* displayed distinct mortality rates. (**b**) In the safety test, WGB-Cory’s pathogenicity was confirmed, with one death observed in the non-vaccinated+CHAL group, while no deaths occurred in the other groups. (**c**) The assessment of anti-PLD antibodies revealed a progressive rise in concentration following vaccination, notably with the vaccinated group demonstrating significantly higher levels (*p* < 0.05). The results for each group were analyzed using a two-way ANOVA. Different number of asterisks within a sampling point mean statistically significant differences (*: *p* < 0.05, ***: *p* < 0.0005, ****: *p* < 0.00005).

A total of 40 mice were used for the LD_50_ experiment to evaluate the challenge with the *C. pseudotuberculosis* field isolate. After the *C. pseudotuberculosis* IP (infection), an approximately 80% mortality rate was observed in Group A (10^8^ CFU/mL) up to 3 days post-challenge (DPC). Groups B (10^7^ CFU/mL) and C (10^6^ CFU/mL) showed severe mortality rates of approximately 80% and 60%, respectively, up to 5 DPC. However, Group D mice, which were challenged with 10^5^ CFU/mL *C. pseudotuberculosis*, did not die until the end of the experiment (Figure 3a). Differences in mortality based on the antigen concentration of the challenge with *C. pseudotuberculosis* were observed in mice; however, no deaths were observed in guinea pigs challenged with the same antigen concentration of bacteria as used in mice.

Based on the antigen concentration determined in guinea pigs, we tested the safety and efficacy of the vaccine candidate WGB-Cory using a total of 30 mice. One mouse in the non-vaccinated+CHAL (Group 2, non-vaccinated, challenged) died on day 4 DPC, whereas no deaths were observed in the vaccine group (Group 1, 10^6^ CFU/mL) and non-vaccinated+non-CHAL (Group 3, PBS) until the end of the experiment (Figure 3b). The challenge dose for guinea pigs was determined at 10^6^ CFU/mL based on the appropriate mortality rate observed in mice. To determine the antigen level of the vaccine candidate to be used, three different vaccine concentrations were analyzed in guinea pigs. After the first vaccination, the anti-PLD antibody concentration gradually increased in the vaccine groups at 4 WPV. After the second vaccination, at 6 WPV, the concentration in Group B was significantly (*p* < 0.005) greater than that in the other vaccine groups (Figure 3c). These data suggest that WGB-cory has safety and efficacy against CLA infection. The guinea pigs in Group B had significantly (*p* < 0.005) greater antibody levels, highlighting the importance of the antigen concentration in vaccine efficacy. Consequently, we confirmed the safety and efficacy of the vaccine candidate material through both guinea pig and mouse models.

### 3.3. Serological Analysis and Clinical Findings in Vaccinated KNBGs

**Figure 4:**
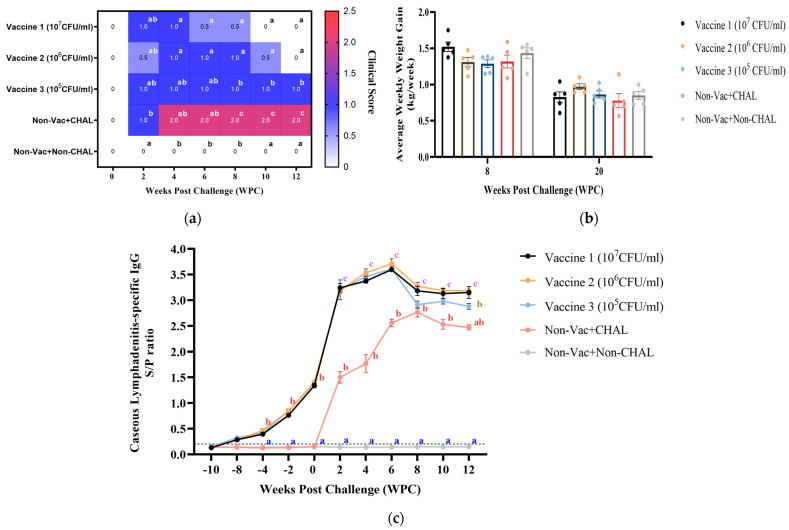
(**a**) Vaccination Safety and Clinical Signs: Following vaccination with various antigen concentrations, KNBGs did not display any adverse effects, with milder clinical signs observed compared to the nonvaccinated+CHAL group at the end of the experiment. (**b**) Average Weekly Weight Gain (AWWG): At 8 WPC, the AWWG for vaccine groups, ranging from 1.29 kg to 1.55 kg, was comparable to that of the non-vaccinated+non-CHAL group. By 20 WPC, vaccine groups exhibited AWWG ranging from 0.78 kg to 0.97 kg, similar to the non-vaccinated+non-CHAL group’s 0.85 kg. (**c**) CLA-Specific Antibody Response: ELISA analysis revealed the absence of CLA-specific IgG in the non-vaccinated+non-CHAL group, while vaccine groups showed significant (*p* < 0.005) increased levels. Although the non-vaccinated+CHAL group peaked with an S/P ratio of 2.766 at 8 WPC, vaccine groups maintained significantly (*p* < 0.005) high levels throughout the experiment. The results for each group were analyzed using a two-way ANOVA. Different letters within a sampling point mean statistically significant differences (*p* < 0.05).

To determine the safety and efficacy of target animals, KNBGs were vaccinated with different concentrations of antigens. After vaccination, side effects such as sudden death and high fever were not observed. The suppuration, necrosis, or cutaneous lesions were not observed at the injection site following vaccination. At 4 WPC, clinical signs such as abscess enlargement, and bursts were observed in the non-vaccinated+CHAL group. In contrast to the vaccinated groups, the non-vaccinated+CHAL group showed significantly (*p* < 0.05) higher than those in the vaccine groups until the end of the experiment (Figure 4a). The impact of vaccination and challenge on body weight changes was observed at designated days. The AWWG of the vaccine groups (vaccine group 1 (10^7^ CFU/mL), vaccine group 2 (10^6^ CFU/mL), and vaccine group 3 (10^5^ CFU/mL)) and the non-vaccinated+non-CHAL group were 1.55 kg, 1.31 kg, 1.29 kg, and 1.43 kg, respectively, at 8 WPC. At 20 WPC, the AWWG of the non-vaccinated+non-CHAL -group was 0.85 kg, whereas those of the vaccine groups were 0.97 kg, 0.87 kg, and 0.78 kg, respectively. No significant differences in body weight were observed between the vaccine group and the non-vaccinated+non-CHAL (Figure 4b). The CLA-specific antibody (total IgG) was measured by ELISA; as expected, the non-vaccinated+non-CHAL group did not show CLA-specific IgG. The vaccine groups showed significantly (*p* < 0.005) increased CLA-specific IgG. Although the non-vaccinated+CHAL group exhibited a mean peak S/P ratio of 2.766 at 8 WPC, the S/P ratio of the vaccine groups remained significantly (*p* < 0.005) higher than that of the non-vaccinated+CHAL (Figure 4c). These findings strongly indicate that WGB-Cory demonstrates both safety and efficacy in KNBGs, emphasizing its potential as a promising candidate for further development.

### 3.4. Bacterial Load and Histopathological Evaluations of the Lymph Nodes

**Figure 5:**
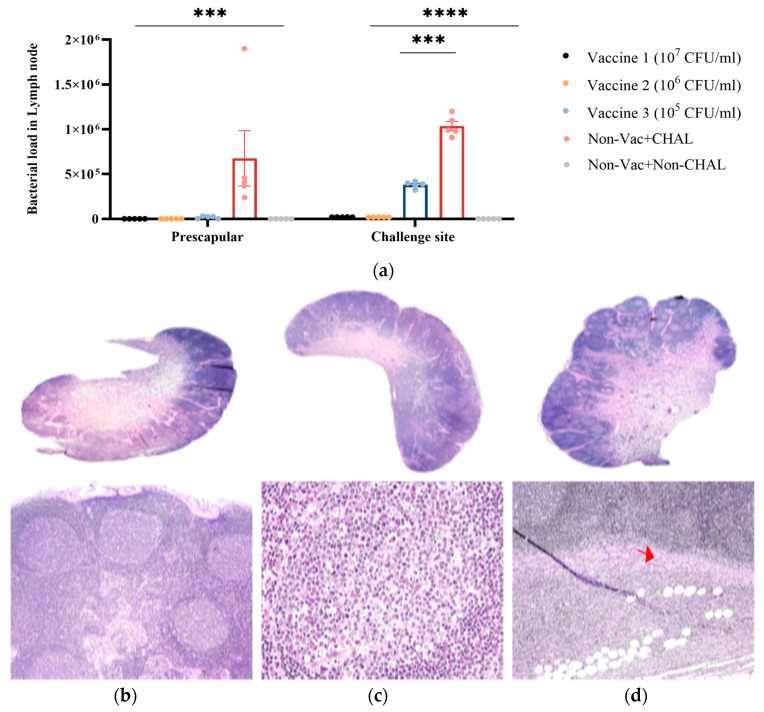
(**a**) Determination of bacterial load in different tissues: Samples were collected from seven lymph node sites. Significant (*p* < 0.005) increases in bacterial loads were detected only in lymph nodes adjacent to the challenge site and in the prescapular lymph nodes, indicative of *C. pseudotuberculosis* infection. (**b**–**d**) Histopathological responses in various lymph node sites. Infiltration of inflammatory cells into adipose tissues and bleeding in the medulla were observed in the non-vaccinated+CHAL. Conversely, the vaccine group showed the absence of lesions specific to CLA infection and exhibited normal histopathological features. (**b**): non-vaccinated+non-CHAL group; (**c**): Vaccinated group; (**d**): non-vaccinated+CHAL group. The results for each group were analyzed using a two-way ANOVA. Different number of asterisks within a sampling point mean statistically significant differences (***: *p* < 0.0005, ****: *p* < 0.00005). The red arrows mark areas of necrosis within the lymph nodes, where inflammatory cell infiltration has been observed around the necrotic regions.

To determine the bacterial load in different tissues, samples were collected from eight lymph node sites. Because of *C. pseudotuberculosis* infection, significantly (*p* < 0.005) greater bacterial loads were detected only in the lymph nodes located next to the site of challenge and in the prescapular lymph nodes (Figure 5a). In contrast, the bacterial loads of the other lymph nodes, such as the retropharyngeal, submandibular, and superficial inguinal lymph nodes, were not observed. Histopathological responses were observed at the prescapular lymph node sites (Figure 5b–d; B: non-vaccinated+non-CHAL, C: Vaccinated group, and D: non-vaccinated+CHAL). The lymph node of the non-vaccinated+non-CHAL group showed an apparently normal lymph node (Figure 5b). In the vaccinated group, lesions specific to CLA infection were not observed (Figure 5c). However, due to *C. pseudotuberculosis* isolate infection, the infiltration of inflammatory cells into adipose tissues and bleeding in the medulla were observed in the non-vaccinated+CHAL group (Figure 5d).

## 4. Discussion

Recently, there has been a significant rise in the demand for KNBGs. Correspondingly, there has been an increased interest in goat diseases, particularly CLA, which has a direct impact on goat prices. Consequently, the need for a vaccine to prevent *C. pseudotuberculosis* infection is also emerging; under these circumstances, it is necessary to develop a CLA vaccine using *C. pseudotuberculosis* strains isolated in Korea. Most CLA research conducted in Korea has focused on the characteristics and morphology of *C. pseudotuberculosis* and eradication programs for this bacterium. In contrast, relatively few studies have addressed vaccine development.

**Figure A1:**
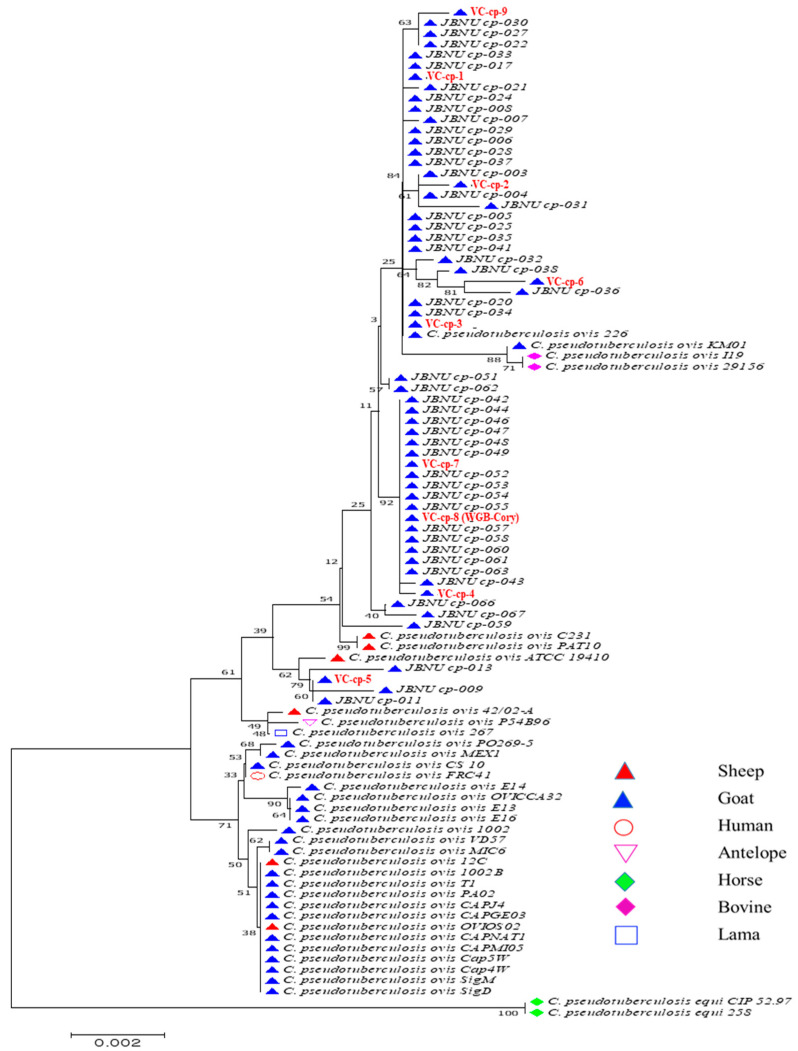
Phylogenetic reconstruction based on concatenated partial sequences of dnaK (520 bp), infB (577 bp), groEL1 (588 bp), leuA (597 bp), and fusA (867 bp) genes. The strains marked in red are candidate bacteria selected from field isolates for vaccine development. The final vaccine strains were chosen through characterization of these field isolates.

The field isolates from Korea were identified by WGS in a previous study [25]. After confirming the molecular characteristics of the field isolates, several evaluations such as virulence genes, growth kinetics of bacteria, and MLST were conducted to assess their possibility as vaccine candidates (Figure A1). All field isolates were classified as biotype *ovis* and were confirmed to contain 17 virulence genes and a PLD gene indicative of pathogenicity.

To develop an inactivated bacterin vaccine, we assessed WGB-Cory through preclinical tests such as identification of molecular characteristics and pathogenicity, confirming its suitability as a vaccine strain. However, conducting preclinical trials has been difficult due to the paucity of literature on CLA infection trials and the lack of studies related to assessing vaccine efficacy. Therefore, we integrated various references to design an experimental model and conducted experiments accordingly. Observing the virulence of WGB-Cory, we conducted pathogenicity evaluations using mice and guinea pig models. However, guinea pigs were not susceptible to *C. pseudotuberculosis*, so pathogenicity was not observed in guinea pigs after the challenge. Nevertheless, it is noteworthy that the CLA-specific antibodies are effectively generated in guinea pig models. Results indicated that while WGB-Cory exhibited significant pathogenicity in the mice, it demonstrated high safety levels following the inactivation process.

Additional experiments were conducted using KNBGs to further explore the effects of the vaccine. The farm where the experiment was conducted had a history of approximately 30–40% CLA infection every year. When the experiment was conducted, CLA infection was confirmed in several individuals. In-vivo experiments were conducted with individuals who did not show clinical symptoms of CLA infection and who did not have CLA antibodies. After a total of two vaccinations, clinical symptoms such as high fever and abscess were not observed. In addition, a significant increase in the levels of antibodies was confirmed in the vaccinated group. After the challenge, the activation of the host immune response was confirmed again with CLA-specific antibodies. Pathology of lymphoid tissue obtained after autopsy confirmed chronic inflammation in the central necrotic core or around the core, a pattern caused by CLA infection, only in the non-vaccinated+CHAL group, and bleeding was also observed in some individuals. These findings observed parallel lesions previously described [30]. In previous studies, vaccination with a combination of recombinant PLD and inactivated whole cells resulted in protection against challenges with virulent bacteria [31,32]. Although the toxoid CLA vaccine is currently marketed in several countries and has demonstrated a certain level of efficacy, our experiments confirmed that there was little difference in efficacy between the toxoid and bacteria CLA vaccines. The disadvantage of the toxoid vaccine involves challenges related to processes such as toxin separation and purification; however, as previously mentioned, the current vaccine was developed as a bacterin vaccine.

In developing the inactivated vaccine, we encountered various challenges. Specifically, the study faced significant impediments, including the limited availability of disease-specific goat antibodies (immune cells) crucial for accurate immune response analysis and a notable deficiency in established experimental protocols and well-characterized animal models tailored for this species. This scarcity of resources and standardized methods severely constrained our ability to comprehensively study and interpret the dynamics of immune responses in vaccinated goats. Despite these challenges, our research enabled us to identify characteristics specific to animal models and gain a preliminary understanding of how they could be applied to vaccine development. This foundational knowledge will guide future refinements in vaccine formulation and administration protocols, ensuring enhanced effectiveness and broader applicability in veterinary practices. This effort will enable the effective execution of subsequent studies aimed at enhancing the efficacy of the bacterin CLA vaccine.

## 5. Conclusions

In this study, we have successfully developed Korea’s first CLA vaccine, named IMMUNIS^®^ CoryVac (tentative name), demonstrating efficacy in Korean native black goats (KNBGs) with confirmed safety. This milestone is on track for imminent domestic approval, with plans for international distribution under consideration. The introduction of an inactivated CLA vaccine is poised to revolutionize domestic black goat farming and offer significant opportunities in both local and global markets. Ongoing and future research endeavors aim to further enhance the vaccine’s effectiveness, fostering continuous advancements in CLA prevention and management.

## Figures and Tables

**Table 1:** Primer sequences, target genes, PCR conditions.

Target Gene	Primers Sequences (5’–3’)	Amplified Size (bp)	Primary Denaturation	Amplification (30 Cycles)			Final Extension
				Secondary Denaturation	Annealing	Extension	
PLD	F; ATGAGGGAGAAAGTTGTTTTA	924	94 °C 5 min	94 °C 30 s	54 °C 30 s	72 °C 90 s	72 °C 10 min
	R; TCACCACGGGTTATCCGC						

## Data Availability

Data supporting the results presented here can be requested by emailing the corresponding author. Data will not be made available publicly or in any format that may violate a study participant’s right to privacy. The whole genome sequencing results used in our vaccine study are registered with NCBI and can be accessed at GenBank: JBBKZZ000000000.1.

## Appendix A

Figure A1, Phylogenetic reconstruction based on concatenated partial sequences of dnaK (520 bp), infB (577 bp), groEL1 (588 bp), leuA (597 bp), and fusA (867 bp) genes; Table A1, Field strains isolated from Korean native black goats (KNBGs).

**Table A1:** Field strains isolated from Korean native black goats (KNBGs).

No.	Strain Name	Vaccine Candidate	Host	Origin	Virulence Gene																	Bacterial Count (CFU/mL, 72 h)
					OppA	OppB	OppC	OppD	OppF	FagA	FagB	FagC	FagD	SigE	SpaC	SodC	PknG	NanH	NrdH	CopC	CpaE	
1	VC *-cp-1	X	KNBG, 13 months	Gokseong-gun	O ^†^	O	O	O	O	O	O	O	O	O	O	O	O	O	O	O	O	8.8 × 10^7^
2	VC-cp-2	X	KNBG, 9 months	Gokseong-gun	O	O	O	O	O	O	O	O	O	O	O	O	O	O	O	O	O	9.2 × 10^7^
3	VC-cp-3	X	KNBG, slaughter	Gochang-gun	O	O	O	O	O	O	O	O	O	O	O	O	O	O	O	O	O	1.2 × 10^8^
4	VC-cp-4	X	KNBG, 18 months	Chongwon-gun	O	O	O	O	O	O	O	O	O	O	O	O	O	O	O	O	O	3.8 × 10^8^
5	VC-cp-5	X	KNBG, slaughter	Gochang-gun	O	O	O	O	O	O	O	O	O	O	O	O	O	O	O	O	O	5.3 × 10^8^
6	VC-cp-6	X	KNBG, slaughter	Gochang-gun	O	O	O	O	O	O	O	O	O	O	O	O	O	O	O	O	O	2.5 × 10^9^
7	VC-cp-7	X	KNBG, 6 months	Namhae-gun	O	O	O	O	O	O	O	O	O	O	O	O	O	O	O	O	O	4.7 × 10^8^
8	VC-cp-8	O	KNBG, 7 months	Gokseong-gun	O	O	O	O	O	O	O	O	O	O	O	O	O	O	O	O	O	4.8 × 10^9^
9	VC-cp-9	X	KNBG, 11 months	Gokseong-gun	O	O	O	O	O	O	O	O	O	O	O	O	O	O	O	O	O	1.5 × 10^9^

*: VC: vaccine candidate: ^†^: virulence gene detected; X: not suitable for vaccine strain; O: Propriate for vaccine strain.
